# Systematic characterization of a non-transgenic A*β*_1–42_ amyloidosis model: synaptic plasticity and memory deficits in female and male mice

**DOI:** 10.1186/s13293-023-00545-4

**Published:** 2023-09-16

**Authors:** Raquel Jiménez-Herrera, Ana Contreras, Souhail Djebari, Jaime Mulero-Franco, Guillermo Iborra-Lázaro, Danko Jeremic, Juan Navarro-López, Lydia Jiménez-Díaz

**Affiliations:** Neurophysiology and Behavior Lab, Biomedical Research Center (CRIB), School of Medicine of Ciudad Real, University of Castilla-La Mancha, 13071 Ciudad Real, Spain

**Keywords:** Alzheimer’s disease, Amyloid-*β*, A*β*_*1–42*_, Spatial memory, LTP, Hippocampus, Sex differences

## Abstract

**Background:**

The amyloid-*β* (A*β*) cascade is one of the most studied theories linked to AD. In multiple models, A*β* accumulation and dyshomeostasis have shown a key role in AD onset, leading to excitatory/inhibitory imbalance, the impairments of synaptic plasticity and oscillatory activity, and memory deficits. Despite the higher prevalence of Alzheimer’s disease (AD) in women compared to men, the possible sex difference is scarcely explored and the information from amyloidosis transgenic mice models is contradictory. Thus, given the lack of data regarding the early stages of amyloidosis in female mice, the aim of this study was to systematically characterize the effect of an intracerebroventricular (*icv.*) injection of A*β*_1–42_ on hippocampal-dependent memory, and on associated activity-dependent synaptic plasticity in the hippocampal CA1–CA3 synapse, in both male and female mice.

**Methods:**

To do so, we evaluated long term potentiation (LTP) with ex vivo electrophysiological recordings as well as encoding and retrieval of spatial (working, short- and long-term) and exploratory habituation memories using Barnes maze and object location, or open field habituation tasks, respectively.

**Results:**

A*β*_1–42_ administration impaired all forms of memory evaluated in this work, regardless of sex. This effect was displayed in a long-lasting manner (up to 17 days post-injection). LTP was inhibited at a postsynaptic level, both in males and females, and a long-term depression (LTD) was induced for the same prolonged period, which could underlie memory deficits.

**Conclusions:**

In conclusion, our results provide further evidence on the shifting of LTP/LTD threshold due to a single *icv*. A*β*_1–42_ injection, which underly cognitive deficits in the early stages of AD. These long-lasting cognitive and functional alterations in males and females validate this model for the study of early amyloidosis in both sexes, thus offering a solid alternative to the inconsistence of amyloidosis transgenic mice models.

**Supplementary Information:**

The online version contains supplementary material available at 10.1186/s13293-023-00545-4.

## Abstract (plain language summary)

This study focuses on investigating how amyloid-*β* (A*β*), a key toxic protein in Alzheimer's disease (AD), impacts memory and functioning of the synapses in both male and female mice.

Our primary objective was to comprehensively understand the impact of A*β*_1–42_, a specific form of A*β*, when introduced into the brain's ventricles, focusing on memory processes associated with the hippocampus, a brain region vital for learning and memory.

Prior research established A*β*'s significance in AD and memory decline. However, despite the higher prevalence of AD in females, the connection between A*β*, memory, and sex differences required further exploration. Furthermore, findings from experiments utilizing A*β* transgenic mice have offered conflicting outcomes. Here, by administering A*β*_1–42_ to male and female mice, we systematically assessed memory using cognitive tasks. Results were consistent: memory deficits were evident in both sexes, persisting for up to 17 days post-injection.

Delving deeper, we explored alterations in synaptic plasticity, a cornerstone of learning and memory. Our investigations unveiled disruptions in long-term potentiation (LTP) and long-term depression (LTD)—essential synaptic processes—in both male and female mice subjected to A*β*_1–42_ injection.

These intriguing findings underscore A*β*_1–42_'s lasting influence on memory and synaptic function, emphasizing its role in early AD-related cognitive decline. Additionally, our study highlights the potential of this experimental model to investigate early AD across sex differences, offering a promising alternative to the existing array A*β* transgenic mouse models and addressing the need for a more consistent investigative framework.

**Supplementary Information:**

The online version contains supplementary material available at 10.1186/s13293-023-00545-4.

## Highlights

- A single *icv*. injection of A*β*_1–42_ disrupts dorsal hippocampal synaptic plasticity and memory processing in both male and female mice.
- A*β*_1–42_ impairs the encoding and retrieval of spatial (working, short- and long-term) and exploratory habituation memories in both male and female subjects.
- A*β*_1–42_ shifts the induction threshold between LTP and LTD in both male and female mice.
- This model for studying early amyloidosis remains valid up to 17 days after a single A*β*_1–42_ administration, regardless of sex.

**Supplementary Information:**

The online version contains supplementary material available at 10.1186/s13293-023-00545-4.

## Background

The role of the hippocampus in learning and memory processes is well established [1, 2]. Several types of memory, such as episodic memory, spatial memory, or contextual fear memory, depend on the hippocampus [3–5]. Spatial navigation studies in rodents have contributed significantly to our understanding of the physiology of the hippocampus, recognizing it as a neural center for spatial mnemonic processing [6]. Spatial memory is essential for the survival of all kind of animals, since it enables the retrieval of object locations and the placement of experiences in a specific environmental context [7]. The encoding of spatial memory relies on the capture of behaviorally relevant spatial cues on a timescale of seconds, which is known as spatial working memory [8]. Moreover, the hippocampus is highly vulnerable to oxidative stress under hypoxia and multiple diseases [9]. This vulnerability contributes to the characteristic memory and learning impairments observed in pathologies like Alzheimer’s disease (AD), addiction or major depression, among others [10, 11].

AD is the leading cause of dementia, affecting over 55 million people worldwide [12]. It typically begins with short-term memory loss, followed by alterations in language and executive functions, as well as visuospatial memory deficits [13]. One of the neuropathological hallmarks of AD is the accumulation of amyloid-*β* (A*β*) plaques, known as senile plaques, in the hippocampus, particularly in the late stages of the disease [14]. A*β* leads to hippocampal dysfunction, including excitatory/inhibitory (E/I) imbalance, impairments of hippocampal oscillatory activity, synaptic plasticity disruption and memory deficits [13, 15, 16]. Electrophysiological data in the early stages of AD, have shown that soluble A*β* oligomers can block hippocampal long-term potentiation (LTP) while enhancing long-term depression (LTD) [17, 18]. These two mechanisms of synaptic strengthening and weakening, respectively, underlie synaptic plasticity. The balance between them is crucial to maintain proper hippocampal functionality, such as learning and memory processes [19]. Indeed, the inhibition of LTP caused by A*β* has been shown to correlate with the memory impairments reported in AD [16, 20–24].

As mentioned above, the accumulation of A*β* in the hippocampus is an important factor for AD development [17] found in both sporadic AD—the most common form of the disease- and familial inherited AD. Many studies use transgenic mice expressing Amyloid Precursor Protein (APP) and accumulating the human A*β* peptide, while other studies induce *tau* hyperphosphorylation by genetic mutation [25]. However, models based on human mutations represent less than 5% of AD cases (inherited AD), since the majority of AD patients have sporadic forms of the disease. Furthermore, the physiopathology of AD begins several decades before the presence of senile plaques, when A*β* is soluble rather than accumulated [26]. Among A*β* fragments, A*β*_1–42_ has a higher propensity to form amyloid fibrils and is the dominant A*β* specie found in the amyloid plaques of AD patients [25, 27]. Moreover, intracerebroventricular (*icv.*) administration of A*β* has been shown to predominantly diffuse to the dorsal hippocampal formation [23], which is specifically related to spatial learning and memory [28, 29]. Therefore, this model appears to be a better option for investigating the early stages of sporadic AD [30]. In this line, previous work from our group has shown an E/I imbalance in male mice following *icv.* injection of A*β*_1–42_, both in vivo and in vitro. This translated into a deficit in LTP induction, a disruption of the correct neural oscillatory synchronization in the hippocampus and, ultimately, an impairment in learning and memory processes [23, 24, 31].

According to the Alzheimer’s Association, nearly two-thirds of AD patients in the US are women [32]. Despite the higher prevalence of AD in women compared to men, most available data comes from studies with male mice [33]. Therefore, it is crucial to include both male and female animals in these types of studies. While a few recent works have done so, the results have been inconsistent, showing either sex-dependent differences [21, 34–37] or no differences [38, 39] depending on the animal model used, specific parameters measured, and/or experimental approaches.

Thus, given the scarcity of data regarding AD models in females, the aim of the present study was to characterize the effects of a single *icv.* injection of A*β*_1–42_ on working, short-term and long-term memory, as well as on synaptic plasticity processes, to provide a solid model for studying early stages of amyloidosis while considering sex differences.

## Materials and methods

### Animals

Female and male C57BL/6 adult mice (12–24 weeks old; 20–30 g) were used (RRID:MGI:5656552; Charles River, USA). The chosen age range falls within the category of mature adult [40]. However, in order to minimize potential age-related effects, data from mice aged 12–18 weeks (3–4 months) and 19–24 weeks (5–6 months) were compared and no significant differences were found (data not shown), warranting their combination. Animals were kept on 12 h light/dark cycles with access to food and water ad libitum. The ambient temperature (21 ± 1 ºC) and humidity (50 ± 7%) were controlled. Mice were housed in same-sex groups of 5 per cage before surgery, and individually afterwards. All experimental procedures were carried out at the same time interval in both female and male mice in order to minimize interference from circadian rhythms.

All experimental procedures were reviewed and approved by the Ethical Committee for Use of Laboratory Animals of the University of Castilla-La Mancha (PR-2022-11-04 and PR-2018-05-11) and conducted according to the European Union guidelines (2010/63/EU) and the Spanish regulations for the use of laboratory animals in chronic experiments (RD 53/2013 on the care of experimental animals: BOE 08/02/2013).

### Surgery for drug injection

The mice were anesthetized with 4% isoflurane (#13400264, ISOFLO, Proyma S.L., Spain) using a calibrated R580S vaporizer (RWD Life Science; flow rate: 0.5 L/min O_2_). Following induction, a constant delivery of 1.5% isoflurane was maintained for anesthesia. Intramuscular buprenorphine (0.01 mg/kg; #062009, BUPRENODALE, Albet, Spain) and a healing cream (Blastoestimulina^®^; Almirall, Spain) were administered as analgesic after surgery, to promote recovery and reduce animal suffering.

**Fig. 1:**
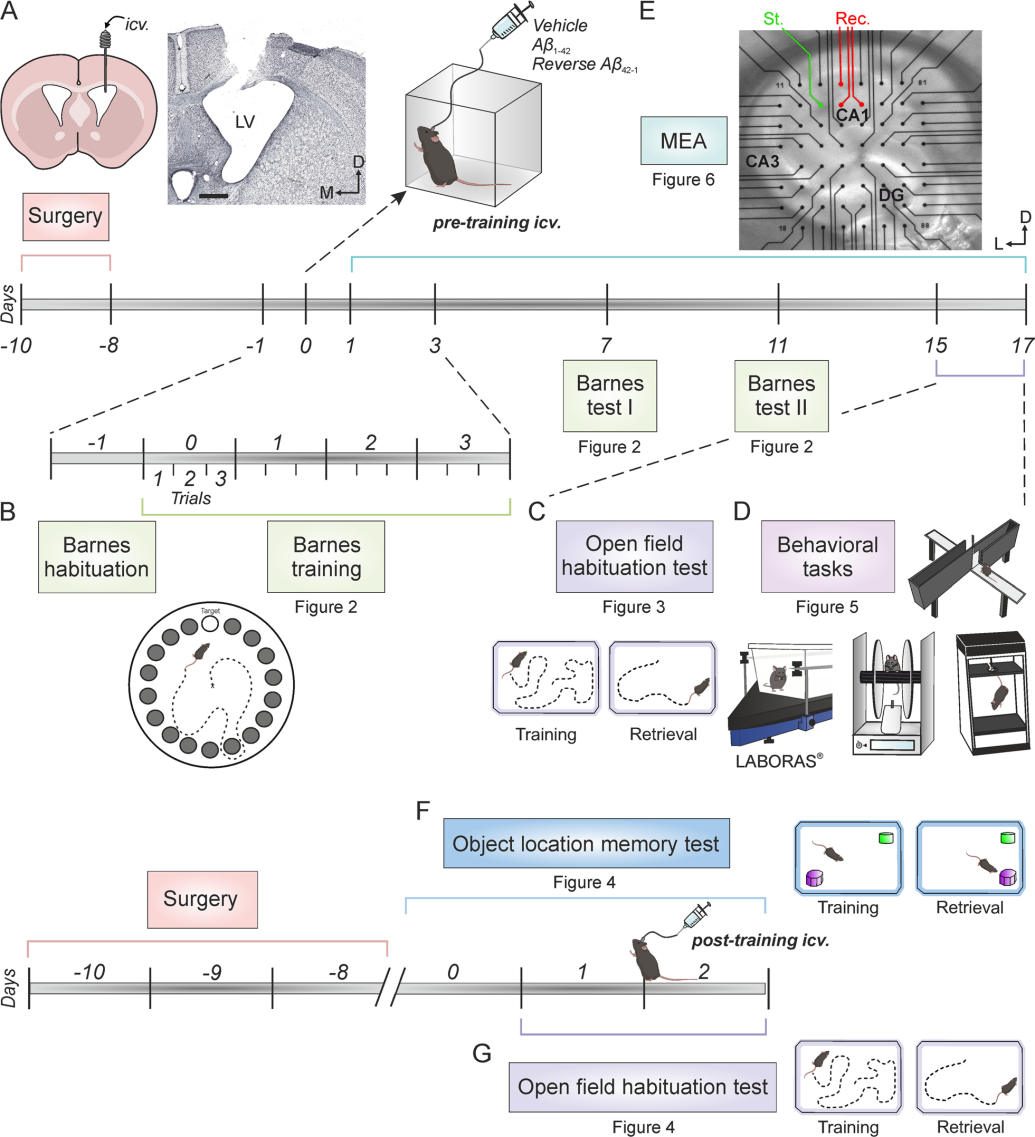
Experimental design showing timeline and corresponding figure numbers. **A** A guide cannula was implanted for *icv*. drug administration on the left ventricle (histologic verification image on the right). A minimum of 8 days after surgery, a single injection of either A*β*_1–42_ or controls (reverse A*β*_42–1_ peptide or vehicle), were administered*.* Scale bars: 500 μm. **B** Habituation phase of Barnes maze task was conducted the day before *icv.* administration (day-1). 1 h after treatment injection (day 0, pre-training *icv. injection*), training in the Barnes maze began, which consisted of 3 trails per day for 4 consecutive days (days 0–3). Two memory tests were conducted, on days 7 and 11 post-injection. **C** The Open field habituation test was conducted on days 15 and 16 post-injection. **D** Finally, mice went through a test battery that included testing of stereotyped and locomotor behaviors by an automated LABORAS^®^ System to assess their overall spontaneous behavior and health, as well as rotarod, elevated plus maze and tail suspension tests (days 15–17). **E** Another cohort of animals was used to evaluate the effect of A*β*_1–42_ on ex vivo hippocampal LTP by multi-electrode arrays (MEAs) electrophysiology (days 1–17 post-injection). Representative location of stimulation (St., green) and recording (Rec., red) electrodes in an hippocampal coronal slice. **F** A different cohort of animals underwent the Object location memory test (OLM) to study the effect of A*β*_1–42_ on memory retrieval. After habituation (day 0), both training and retrieval (OLM1) sessions were conducted on day 1. On day 2, animals were *icv.* injected with the corresponding treatment (post-training injection) and, 1 h later, another retrieval session (OLM2) was performed. **G** The same animals also underwent the Open field habituation test, with post-training *icv*. treatment. D, dorsal; DG, dentate gyrus; *icv*., intracerebroventricular; L, lateral; V, ventricle; M, medial

As described elsewhere [24], for *icv*. administration of A*β*, animals were implanted with a blunted, stainless steel, 26-G guide cannula (Plastics One, USA) targeting the left ventricle (1 mm lateral and 0.5 mm posterior to bregma; depth from brain surface, 1.8 mm) [41]. The final position of the cannula was determined by Nissl staining (Fig. 1A).

Mice were allowed at least a week for recovery before any experimental procedures were conducted. Once fully recovered, freely moving animals received a single 3 μl *icv.* injection of either 1µg/µL A*β*_1–42_, A*β*_42–1_ (as a reverse peptide control) or vehicle (control) through an injection cannula at a rate of 0.5 μL/min. For this purpose, the injection cannula was inserted into the guide cannula protruding 0.5 mm into the ventricle and attached to a Hamilton syringe. The single *icv.* injection was performed on Day 0, i.e. before any behavioral testing, in order to evaluate the effect of the treatment on memory encoding (pre-training *icv.* injections, see Fig. 1A for details of the experimental design). To study the effect of the peptide on memory retrieval, the single *icv.* injection was conducted after training (i.e. on Day 2) of the corresponding behavioral test (post-training *icv.* injections, Fig. 1F, G). Drug concentrations were chosen based on previous studies [23, 24, 31]. All drugs were dissolved in phosphate-buffered saline (PBS) and purchased from Bachem (#4014447 and #4027991, respectively; Switzerland). To verify the clearance of the amyloid peptide, western blot analysis was carried out and the levels of hippocampal A*β*_1–42_ protein were measured at three time points: 1 h, 24 h and 17 days post-*icv*. administration (see Additional file 1). Mice were routinely handled to minimize stress throughout the experimental procedures.

### Barnes maze

To evaluate hippocampal-dependent working, short-term and long-term spatial memory, the Barnes maze (LE851BSW, Panlab, Spain) was used [42]. The maze consists of a rotating platform disk (92 cm in diameter) with 20 escape holes (5 cm each) positioned around its periphery, elevated 1 m above the floor. Spatial cues (circles and squares of different colors) and mildly aversive white noise were used.

The protocol used was adapted from Suarez et al. [43]. Briefly, each trial began with the mouse inside a starting cylinder (8 × 12.5 cm) positioned at the center of the maze. After a 10 s period, the starting cylinder was removed, and the white noise was initiated. Between each trial, the maze was cleaned with 70% ethanol to dissipate odor cues. As shown in Fig. 1B, the protocol consisted of a habituation trial conducted one day prior to the *icv.* injection (Day-1), followed by 4 days of training with three trials each, starting from the day of treatment administration (Days 0–3), and two memory tests (Days 7 and 11). During the habituation day, mice were allowed to explore the maze for 90 s or until they found the goal box (17.5 × 7.5 × 8 cm) attached to one of the holes. If the time limit was reached, the animal was gently guided to the goal box. In both cases, mice remained in the box for 1 min before being returned to their cages. Afterwards, training was carried out over four consecutive days, with three trials per day and a 15 min interval between trials. During each trial, mice were allowed to explore the maze for 3 min or until they found the escape hole, which was changed daily. Latency to find the box, errors made, and distance traveled were measured. Finally, a single 90 s trial was performed each memory testing day, during which no escape hole was available. Latency and distance to the target of the latest training day were measured. During both training and tests sessions, the number of errors and the number of quadrant crosses were used to categorize animals based on their search strategy: Spatial or direct (defined as 0 quadrant crosses and < 3 errors), serial (defined as < 3 quadrant crosses and a sequential order of hole visits), and random (if the movement history did not satisfy the conditions for spatial or serial) [44, 45]. All sessions were recorded and analyzed with Barnes-Smart video tracking software (Panlab, Spain).

### Open field habituation task

On days 15 and 16 post-*icv.* injection (Fig. 1C), an open field (OF) habituation task [46] was conducted in order to evaluate a non-associative hippocampal-dependent learning process, such as exploratory habituation to a novel environment [47] in the same cohort of mice tested in the Barnes Maze. Briefly, mice were exposed to an OF on two consecutive days, and the change in exploration after re-exposure was measured. On the training day (OF1), mice were placed in the center of a square acrylic box (23.5 × 17.5 × 4 cm plexiglas base arena; 26.5 × 21 × 10 cm top) and allowed to freely explore the environment for 15 min. 24 h later, on the retrieval day (OF2), mice were re-exposed to the same environment. Exploratory behavior was recorded using a LABORAS^®^ apparatus (*Laboratory Animal Behavior Observation Registration and Analysis System*; Metris, Netherlands), which captures mechanical vibrations generated by the movements of the animals and convert them into electrical signals through a sensing platform positioned beneath the cage.

Additionally, a second cohort of animals was used to specifically evaluate memory retrieval using the same habituation task described above. In this case, the *icv*. injection was administered between the training day (OF1) and the retention day (OF2), allowing for an investigation of the effects of the injection on memory recall during the retrieval session (Fig. 1G).

### Object location memory

To further evaluate memory retrieval in a spatial hippocampal-dependent task, animals from the second cohort also underwent the object location memory (OLM) test (Fig. 1F), following a protocol adapted from Zhang et al. [48].

Initially, the mice were habituated to a 49 × 49 cm square box for a period of 90 s. Visual cues were positioned within the room to provide spatial context. The following day, a training session was carried out in which two objects were placed within the chamber, and mice were given 10 min to freely explore the objects. 5 h after the training session, a memory test (OLM1) was performed, lasting 5 min. During this test, one of the objects was moved to a novel location within the box. 24 h later, mice were *icv.* injected with the corresponding treatment, and 1 h post-*icv*. injection, another memory test was conducted (OLM2) to evaluate retrieval, moving the object to a third location. Between each mouse challenge, both box and objects were cleaned with 70% ethanol to remove any residual odor cues.

The time spent exploring each object was measured, and discrimination index (DI) was calculated for both OLM1 and OLM2 with the following formula: (Time exploring moved object − time exploring unmoved object)/Total exploration time. Animals with a total exploration time shorter than 5 s were excluded from the analysis.

### Spontaneous behaviors

Between days 15 and 17 post-injection, mice went through a battery of behavioral tests (stereotyped and locomotion, rotarod performance, elevated plus maze and tail suspension tests) to assess their overall state and spontaneous behaviors (Fig. 1D).

#### Laboratory animal behavior observation registration and analysis system (LABORAS^®^) for stereotyped and locomotion behavioral testing

Mice were placed in a rectangular LABORAS^®^ cage for a single 15-min trial, as previously described [46] to evaluate stereotyped behaviors and locomotion. Grooming behavior was used as a measurement of stress-related behavior [49]. Locomotion, climbing, and rearing behaviors were used as measurements of locomotor activity [50]. All data were digitized and analyzed using the LABORAS^®^ software (Metris, The Netherlands).

#### Rotarod performance test

The rotarod apparatus (LE 8500, Panlab, Spain), consisting of a 30 mm diameter black striated rod positioned 20 cm above the floor, was used to measure coordination and motor function. Initially, the mice were trained to stay for 1 min on the rod at constant low-speed (6 rpm) rotation. On the following day, 5 consecutive trials were carried out, with the rod accelerating from 4 to 40 rpm over a 2 min period, and mice’s time to fall off the rod was recorded.

#### Elevated plus maze

With the aim to assess anxiety-like behaviors [51], the elevated plus maze (LE 842, Panlab, Spain) was used. It consists of a cross-shaped methacrylate platform with two open arms (65 × 6 cm) without walls and two enclosed arms (65 × 6 cm) with 15-cm-high opaque walls, mounted 90° to one another with a central platform (6.3 × 6.3 cm) and raised 40 cm above the floor. Mice were placed into one of the open arms and allowed to freely explore the maze for a single 5 min session. The number of entries into the open arms and the time spent in those arms were measured as indicators of anxiety-like behavior. Additionally, the number of entries into the closed arms and the total entries (open + closed arms) were recorded as a further measurement of locomotor activity [52]

#### Tail suspension test

To assess depression-like behaviors, the tail suspension test was performed [53]. The tail suspension apparatus (BIO-TST5, Bioseb, US) consists of three PVC chambers (50 × 15 × 30 cm each) in which the animals were hung by their tails approximately 10 cm away from the ground for 6 min. The immobility time was recorded using strain sensors. All data were digitized and analyzed using the BIO-TST5 software (Bioseb, USA).

### Ex vivo field EPSP (fEPSP) recordings

Coronal hippocampal slices were prepared as previously described [46]. In summary, the animals were deeply anesthetized with halothane (Fluothane, AstraZeneca, UK) and decapitated. The brain was immediately removed and rapidly immersed in oxygenated (95% O_2_–5% CO_2_) ice-cold "cutting" solution containing (in mmol/L; all from Sigma, US): 87 NaCl (#S9888), 10 glucose (#G8270), 75 sucrose (#84100), 1.25 NaH_2_PO_4_ (#S8282), 3 C_3_H_3_NaO_3_ (#P2256), 0.98 C_6_H_7_NaO_6_ (#11140), 25 NaHCO_3_ (#S6014), 2.5 KCl (#P3911), 0.37 CaCl_2_ (#499609), 3.28 MgCl_2_ (#208337). The brain was trimmed and mounted on the stage of a vibratome (7000smz-2; Campden Instruments, UK) in a way that allowed the blade to cut through hemispheres at an angle of 20–30° from their horizontal planes. Coronal slices (300 µm thick) containing the dorsal hippocampus were then incubated for at least 1.5 h at room temperature (22 °C) in oxygenated artificial cerebrospinal fluid (aCSF) containing (in mmol/L; all from Sigma, US): 125.99 NaCl (#S9888), 3 KCl (#P3911), 1.8 CaCl_2_ (#499609), 1.5 MgCl_2_ (#208337), 25.99 NaHCO_3_ (#S6014), 10 glucose (#G8270), and 1.2 NaH_2_PO_4_ (#S8282).

For electrophysiological recordings, two set-ups consisting of a multi-electrode array (MEA2100-Mini-System) pre-amplifier and a filter amplifier (gain 1100 × or 550 ×) were run in parallel using a data acquisition card governed by MC_Experimenter V2.20.0 software. A single slice was transferred to each MEA recording chamber (MEA60; Multi Channel Systems, Reutlingen, Germany), which was continually perfused with aCSF (flow rate 2 mL/min) and kept at 32 °C. The MEA, positioned on the platform of an inverted MEA-VMTC-1 video microscope, consisted of 60 extracellular electrodes (inter-electrode distance: 200 μm). Each individual electrode from the array could be used either as a recording or as a stimulation electrode. A nylon mesh was positioned above the slice to ensure good electrical contact between the slice surface and the electrode array. Stimulation was achieved with a stimulus generator unit integrated in the headstage (Multi Channel Systems, Germany) by applying biphasic current pulses to one electrode of the array (S1) located in the Schaffer Collateral pathway of the hippocampus. Field excitatory postsynaptic potentials (fEPSPs) were recorded in the *stratum radiatum* of the CA1 subfield by all the remaining electrodes of the array simultaneously (Fig. 1E). A second electrode of the array (S2) was used to stimulate an independent pathway, as control of synaptic transmission.

After an equilibration period of at least 20 min inside the MEA chamber, basal synaptic transmission was examined using input/output (I/O) curves. Two stimuli of increasing intensities (0.02–0.4 mA) were applied at a 40 ms interstimulus interval. After I/O, pulse intensity was adjusted to ≈ 40% of the intensity required to evoke a maximum fEPSP response. To address a typical short-term plasticity phenomenon at a presynaptic level, the paired-pulse facilitation (PPF) protocol was used. Pairs of stimuli were delivered at different interstimulus intervals (10, 20, 40, 100, 200, 500 ms). For LTP induction, a high-frequency stimulation (HFS) protocol was used, consisting of five 1-s-long 100-Hz trains delivered at a 30 s intertrain interval. Baseline (BL) values of fEPSPs amplitude recorded at the CA3-CA1 synapse were collected for 15 min before LTP induction. Following the HFS, fEPSPs were recorded for 60 min to evaluate LTP induction. To pair it with the behavioral tasks, electrophysiology recordings were carried out 1–17 days after the *icv.* injection of A*β*_1–42_ or controls: A*β*_42–1_ and vehicle. To distinguish between acute and long-term effects of the treatments on LTP, the recordings were grouped into two time intervals: 24–48 h post-injections and 3–17 days post-injection.

Data was analyzed with the Multichannel Analyzer software (V 2.20.0). As synaptic responses were not contaminated by population spikes, the amplitude (i.e., the peak-to-peak value in mV during the rise-time period) of successively evoked fEPSPs was measured. All values are represented as mean ± SEM with *n* indicating number of slices. Within each slice, data from 3 different recording electrodes were used.

### Statistical analysis

Data was represented as the mean ± SEM and analyzed by three- or two-way ANOVA, followed by Tukey’s *post-hoc* analysis. When comparing only two groups, Student t test was used. Statistical significance was set at *p* < 0.05. All analyses were performed using SPSS software v.24 (RRID:SCR_002865; IBM, USA) and GraphPad Prism software v.8.3.1 (RRID:SCR_002798; Dotmatics, USA). Final figures were prepared using CorelDraw X8 Software (RRID:SCR_014235; Corel Corporation, Canada).

## Results

Firstly, we wondered whether a single *icv.* injection of A*β*_1–42_ would affect the encoding and retrieval of spatial and habituation learning and memory in a sex-specific manner. Thus, female and male mice underwent a protocol to measure spatial learning and memory using a Barnes maze or an OLM test, as well as an exploratory habituation protocol using an OF to assess this type of non-associative memory.

### A*β*_1–42_ impairs spatial learning and memory encoding in both female and male mice

**Fig. 2:**
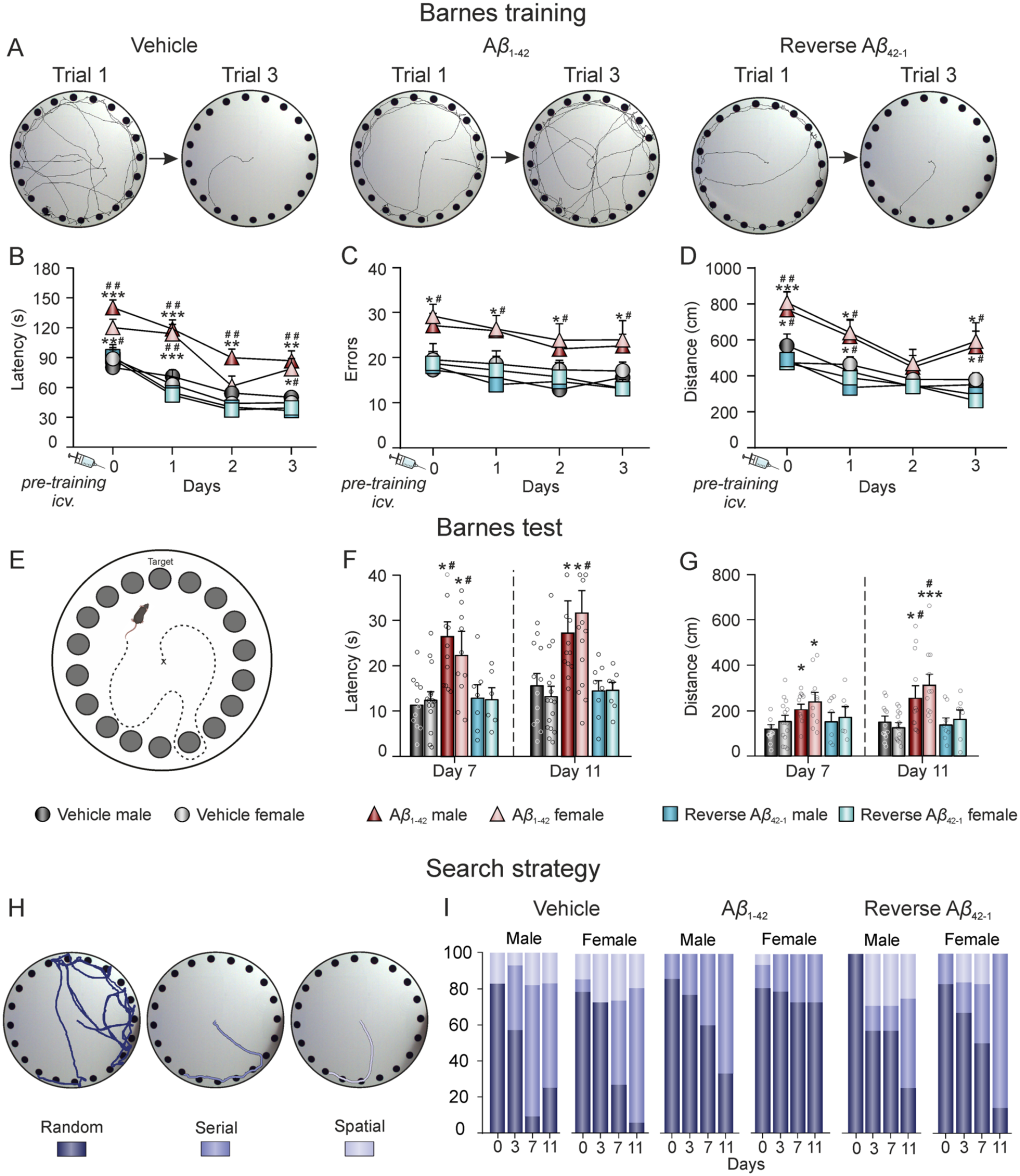
A*β*_1–42_ impairs spatial learning and memory encoding in both female and male mice. **A** Representative traces of the path traveled during the first and last trial of the last training day (day 3) for A*β*_1–42_ and controls: vehicle and reverse A*β*_42–1_ peptide. **B–D** Escape latency (**B**; in s), number of errors (**C**) and distance traveled (**D**; in cm) during the four training days. Data is expressed as the mean ± SEM of the 3 trials per day. **E** Overview image of the test phase on the Barnes maze, with all holes closed. **F** Latency (in s) to reach the target hole of the latest training day for the first time, during the two test sessions. **G** Distance traveled (in cm) during the two test sessions. **H** Representative traces of the three possible search strategies: random, serial, and spatial. **I** Ratio of the use of each search strategy for all the experimental groups during training (Days 0 and 3) and tests (Days 7 and 11) sessions. Stacked bars are normalized so that the sum of the three strategies each day is 100%. N vehicles: males = 14 and females = 15; N A*β*_1–42_: males = 14 and females = 16; N reverse A*β*_42–1_ males = 7 and females = 6. A*β*, Amyloid-*β*; cm, centimeters; s, seconds. *p < 0.05, **p < 0.01, *** *p* < 0.001 *vs*. vehicle of the corresponding sex; # *p* < 0.05, ## *p* < 0.01 *vs*. A*β*_42–1_ of the corresponding sex

To evaluate spatial memory encoding, we employed the Barnes Maze task, where animals performed 3 trials per day over 4 consecutive days (days 0–3), starting 1 h after *icv.* injections (performed in day 0, Fig. 1) of either A*β*_1–42_ or both controls: vehicle and reverse A*β*_42–1_ peptide. The latency to find the open hole was measured in the first and last trial of each day (Fig. 2A) to assess working memory [43]. No differences between males and females were found within each treatment group (sex effect: F_(1,118)_ = 0.096, *p* = 0.757). Nevertheless, our data showed a significant treatment (F_(2,118)_ = 18.147, *p* < 0.001) and time (F_(1,118)_ = 42.742, *p* < 0.001) effects when comparing the latency to find the escape hole in the first and last trial from each training session, proving that both vehicle (male, n = 14; female, n = 15) and A*β*_42–1_ (male, n = 7; female, n = 6) groups had a normal spatial working memory, while the A*β*_1–42_ group (male, n = 14; female, n = 16) showed a deterioration in the formation of this kind of memory, irrespective of sex. Furthermore, the evolution of different parameters measured over the four training days allowed us to evaluate short-term memory, since animals should remember how to perform the task and therefore latencies would decrease over time, even if the target hole was changed. Accordingly, our data showed treatment and time effects for latency (Fig. 2B; F_(2,254)_ = 29.623, *p* < 0.001 and F_(3,254)_ = 22.861, *p* < 0.001, respectively), number of errors (Fig. 2C; F_(2,241)_ = 29.358, *p* < 0.001 and F_(3,241)_ = 3.365, *p* = 0.019, respectively) and distance traveled (Fig. 2D; F_(2,231)_ = 26.673, *p* < 0.001 and F_(3,231)_ = 8.508, *p* < 0.001, respectively). *Post-hoc* analysis revealed that A*β*_1–42_ disrupted spatial learning in both female and male animals, since neither latency (F_(1,254)_ = 0.212, *p* = 0.646), errors (F_(1,241)_ = 1.046, *p* = 0.307), nor distance (F_(1,231)_ = 0.095, *p* = 0.758) showed a sex effect. Finally, long-term memory was assessed through two memory tests conducted on days 7 and 11 post-injection, during which all holes were closed (Fig. 2E). Latency to reach the target hole of the last training day (day 3), as well as distance traveled, were quantified. Our results showed a statistically significant difference in latency (Fig. 2F; treatment effect: F_(2,113)_ = 15.894, *p* < 0.001; sex effect: F_(1,113)_ = 0.002, *p* = 0.967) and distance traveled (Fig. 2G; treatment effect: F_(2,231)_ = 26.673, *p* < 0.001; sex effect: F_(1,231)_ = 0.095, *p* = 0.758). Once again, *post-hoc* analysis revealed that the differences were due to a worse performance of the A*β*_1–42_-treated mice.

Furthermore, navigation strategy during the training and tests sessions was studied for all treatment groups. Although the Barnes maze is considered to encourage allocentric strategies due to the use of distal visual cues [54], different searching strategies, that reflect different levels of learning, could be used by the animals: random, serial and spatial (Fig. 2H). Our data showed that all groups initially employed the random strategy, which decreased across days (Fig. 2I; F_(2.85,182.414)_ = 12.535, *p* < 0.001, Geisser-Greenhouse’s correction). However, a significant treatment effect was found (F_(5.7,182.414)_ = 2.575, *p* = 0.022, Geisser-Greenhouse’s correction), while no sex effect was observed (F_(2.85,182.414)_ = 0.037, *p* = 0.989, Geisser-Greenhouse’s correction). This indicates that both male and female mice treated with A*β*_1–42_ continued to rely on the random strategy compared to the control groups, which is consistent with the impaired memory observed in the A*β*_1–42_ group.

Thus, these data suggest an impairment in the encoding of hippocampal-dependent spatial working, short- and long-term memory and learning processes induced by A*β*_1–42_ in both sexes. In contrast, same concentrations of the control reverse peptide, A*β*_42–1_, did not differ from the control vehicle, suggesting that the decline induced by A*β*_1–42_ was therefore specific.

### A*β*_1–42_ impairs the encoding of exploratory habituation memory in both female and male mice

**Fig. 3:**
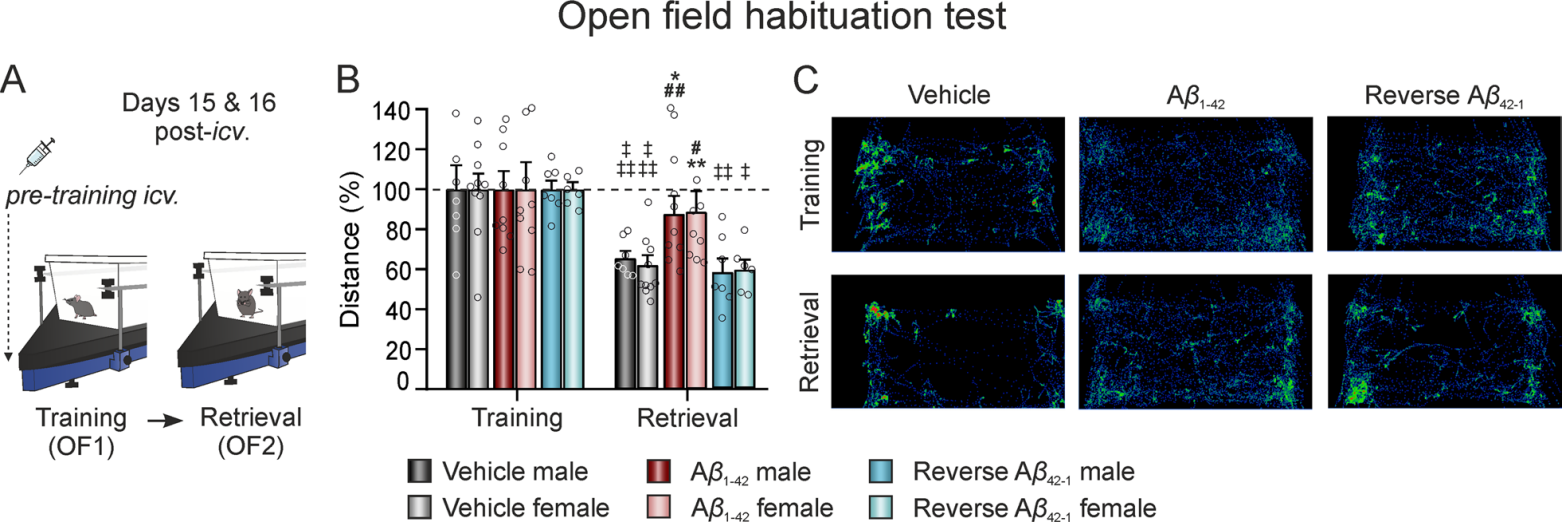
A*β*_1–42_ administration similarly alters non-associative exploratory habituation memory encoding in both female and male mice.** A** An OF habituation test was carried out by submitting the animals to the same OF arena twice, on consecutive days 15 and 16 post-*icv.* injection. **B** Total distance traveled during the two OF sessions (training -OF1- and retrieval -OF2- sessions). Data is expressed as the percentage (%) of the distance traveled in the training session (OF1). **C** Examples of mice movement tracked during OF1 and OF2 for the different treatment groups. N vehicles: males = 7 and females = 12; N A*β*_1–42_: males = 9 and females = 10; N reverse A*β*_42–1_: males = 7 and females = 6. A*β*, Amyloid-*β*; *icv*., intracerebroventricular; OF, open field. * *p* < 0.05, ** *p* < 0.01 *vs*. vehicle of the corresponding sex; # *p* < 0.05, ## *p* < 0.01 *vs*. A*β*_42–1_ of the corresponding sex; ‡ *p* < 0.05, ‡‡ *p* < 0.01, ‡‡‡ *p* < 0.001 *vs*. OF1

Afterwards, exploratory habituation memory encoding was assessed using an OF habituation task on days 15 and 16 post-*icv.* injection (Fig. 3A). Data showed no significant differences in exploration between the groups during the training session (OF1). However, when comparing OF1 and the retrieval session (OF2) (Fig. 3B), significant treatment (F_(2,84)_ = 3.464, *p* = 0.0358) and time effects (F_(1,84)_ = 34.95, *p* < 0.0001) were observed, with no sex effect (F_(1,84)_ = 0.0023, *p* = 0.9614), showing a decrease in exploration in both controls, vehicle (Male, n = 7; Female, n = 12) and A*β*_42–1_ (Male, n = 7; Female, n = 6) treated animals, regardless of sex, proving that they were able to remember the arena. Conversely, A*β*_1–42_-treated mice (Male, n = 9; Female, n = 10) performed slightly better without reaching significance, suggesting some deterioration of memory encoding in this group of animals. Moreover, during the retrieval session (OF2), a significant treatment effect was found (Fig. 3B; F_(2,42)_ = 9.342, *p* = 0.0004) without a sex effect (F_(1,42)_ = 0.0063, *p* = 0.9369). *Post-hoc* analyses revealed that both male and female A*β*_1–42_ mice traveled a longer distance during this session compared to the vehicle and A*β*_42–1_ control groups, as illustrated in Fig. 3C. Hence, both results show that A*β*_1–42_ also impairs the ability to generate non-associative habituation memory even 2 weeks after treatment.

### A*β*_1–42_ impairs spatial and habituation memory retrieval in both female and male mice

**Fig. 4:**
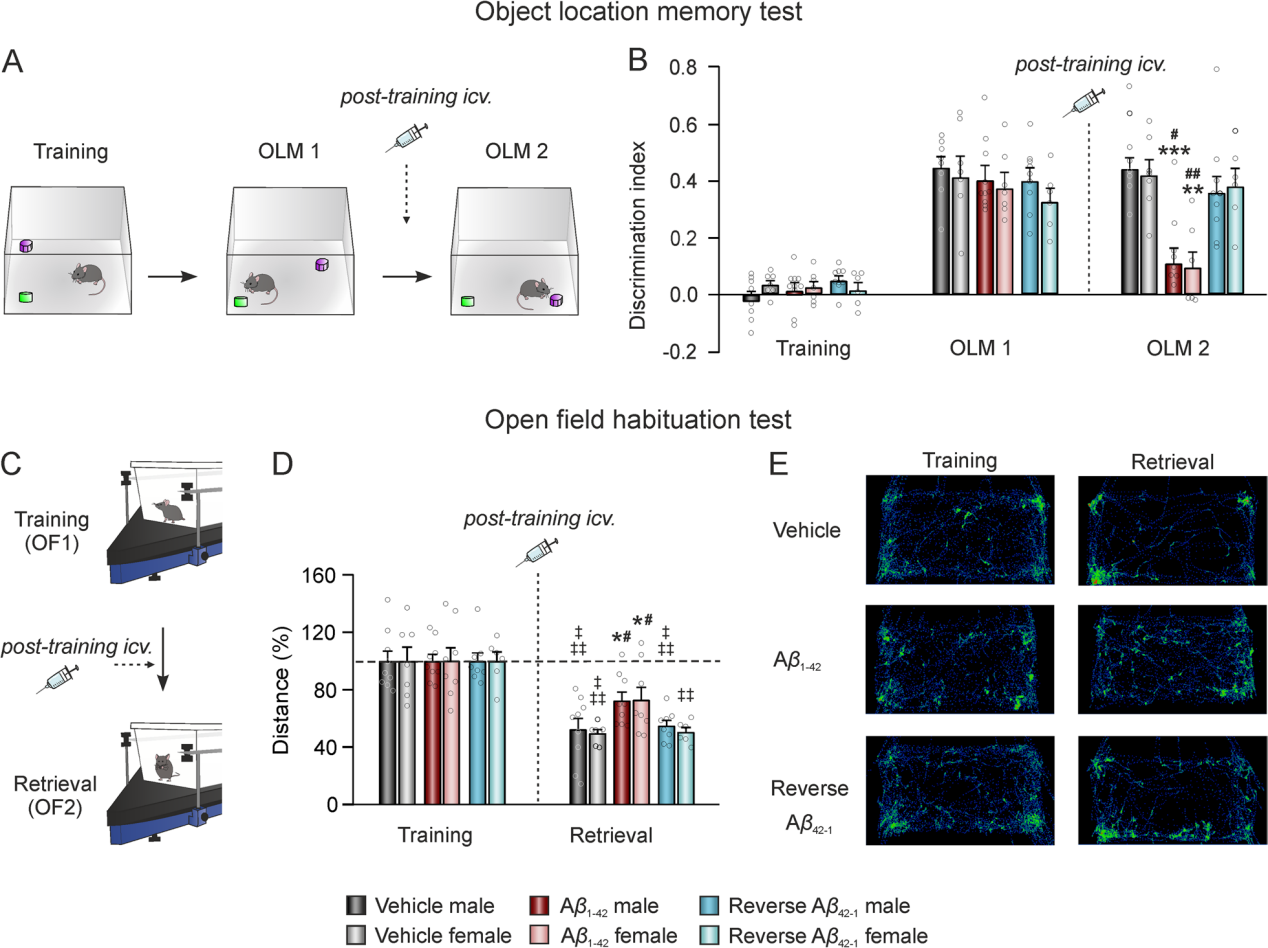
A*β*_1–42_ administration also alters spatial and non-associative memory retrieval in both female and male mice.** A** An OLM test was performed, changing the location of one object between the training and each memory test (OLM1 and OLM2). Treatment was administered *icv.* between OLM1 and OLM2 to evaluate memory retrieval. **B** Discrimination index during the training, OLM1, and OLM2 sessions. Data is expressed as the mean ± SEM. **C** An OF habituation test was carried out by submitting the animals to the same OF arena twice, administering the *icv*. injection between the training and the retrieval sessions. **D** Total distance traveled during the two OF sessions (training -OF1- and retrieval -OF2- sessions). Data is expressed as the percentage (%) of the distance traveled in the training session (OF1). **E** Examples of mice movement tracked during OF1 and OF2 for the different treatment groups. N vehicles: males = 8–9 and females = 7; N A*β*_1–42_: males = 9–10 and females = 7–8; N reverse A*β*_42–1_: males = 8–9 and females = 6. A*β*, Amyloid-*β*; *icv*., intracerebroventricular; OF, open field; OLM, object location memory. *p < 0.05, **p < 0.01, ***p < 0.001 *vs*. vehicle of the corresponding sex; #*p* < 0.05, ##*p* < 0.01 *vs*. reverse A*β*_42–1_ of the corresponding sex; ‡‡*p* < 0.01, ‡‡‡*p* < 0.001 *vs*. OF1

The above experiments proved the deleterious effect of a single injection of A*β*_1–42_ on the encoding phase of memory when delivered before learning each task. To investigate the impact on memory retrieval, a second set of experiments was conducted. In this set, A*β*_1–42_ was injected after the learning phase to evaluate both spatial and exploratory habituation memories using an OLM test and the OF habituation task, respectively (Fig. 4).

Data from the OLM training session showed that all the animals spent equal amount of time exploring both objects (DI ≈ 0 accounts for no preference for a specific object which could have influenced the later results). During OLM1, no differences in the DI due to either sex (F_(1,41)_ = 1.4, *p* = 0.2436) or treatment (F_(2,41)_ = 1.011, *p* = 0.3727) were found, indicating that all naïve animals had proper spatial memory encoding. However, during OLM2, conducted 1 h after treatment, two-way ANOVA showed a significant treatment effect (F_(2,40)_ = 23.26, *p* < 0.0001) regardless of sex (F_(1,40)_ = 0.0003, *p* = 0.9855). *Post-hoc* analysis revealed that A*β*_1–42_ treated mice (Male, n = 10; Female, n = 7) exhibited a lower DI compared to both vehicle (Male, n = 8; Female, n = 7) and A*β*_42–1_ mice (Male, n = 9; Female, n = 6) of the corresponding sex (Fig. 4A, B).

Furthermore, the OF habituation task (Fig. 4C–E) showed a significant treatment (F_(2,82)_ = 3.277, *p* = 0.0427) and time effects (F_(1,82)_ = 108.9, *p* < 0.0001), with no sex effect (F_(1,82)_ = 0.07784, *p* = 0.7809), when comparing the pre-treatment OF1 with the post-treatment OF2. Resembling earlier outcomes, there was a decrease in exploration in both vehicle (Male, n = 9; Female, n = 7) and reverse A*β*_42–1_ (Male, n = 8; Female, n = 6) animals, indicating memory retrieval of the arena. In contrast, A*β*_1–42_-treated mice (Male, n = 9; Female, n = 8) performed slightly better without reaching significance, suggesting some deterioration of memory retrieval for this group. Moreover, during the retrieval session (OF2), a significant treatment effect was found (F_(2,41)_ = 7.647, *p* = 0.0015), with no sex effect (F_(1,41)_ = 0.1816, *p* = 0.6722). *Post-hoc* analyses revealed that both male and female A*β*_1–42_ mice traveled a longer distance during this session compared to vehicle and reverse A*β*_42–1_ control groups, as illustrated in Fig. 4D, E.

These results collectively show that A*β*_1–42_ also impairs both spatial and non-associative habituation memory when administered after learning, thus affecting both encoding and retrieval processes.

### Memory impairments were not due to health or locomotor disfunction

**Fig. 5:**
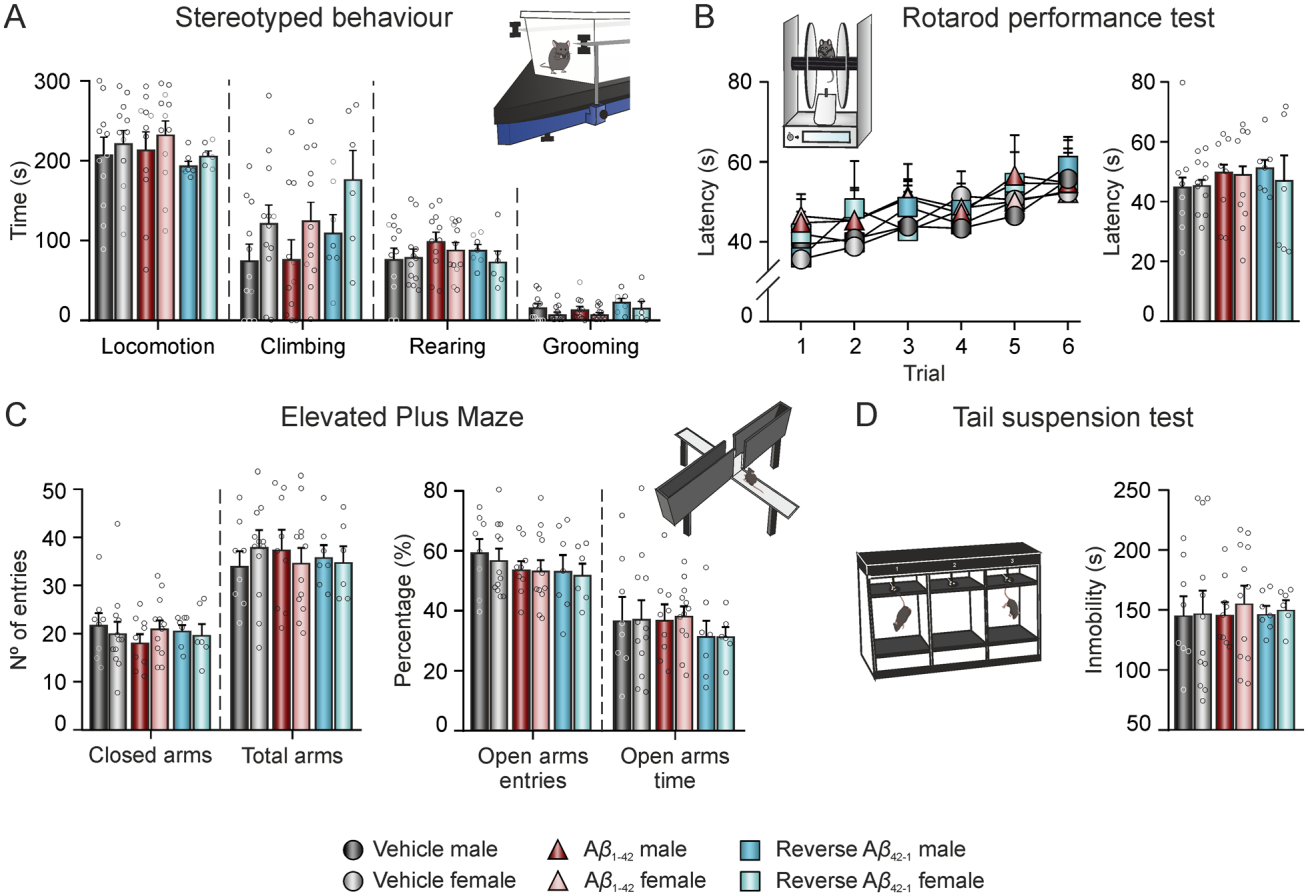
A*β*_1–42_ administration does not induce alterations in locomotor activity, anxiety, and depression-like behavior. Behavioral tasks to evaluate general health state were carried out on days 15–17 post-*icv.* injection. **A** Stereotyped behaviors were assessed using a LABORAS^®^ system, measuring the time (in s) spent performing each type of activity (locomotion, climbing, rearing, and grooming). **B** Latency (in s) to fall off the Rotarod during the six trials (left) and the whole session (right). **C** Number of entries in closed and total arms were used as measure of locomotor activity (left), while anxiety levels were assessed by the percentage (%) of entries and time spent on open arms in an elevated plus maze (right). **D** Depression-like behavior was assessed by measuring the immobility time (in s) during a single session in the tail suspension test. N vehicles: males = 8–11 and females = 11–12; N A*β*_1–42_: males = 8–11 and females = 9–12; N reverse A*β*_42–1_: males = 7 and females = 6). A*β*, Amyloid-*β*; s, seconds

Mice underwent a battery of behavioral tests in order to assess general health conditions and confirm that the impairments observed in the encoding and retrieval of hippocampal-dependent spatial and habituation memory were due to specific hippocampal alterations triggered by A*β*_1–42_ injection. Using the LABORAS^®^ to assess stereotyped behaviors, data showed that all groups (vehicle male, n = 11; vehicle female, n = 12; A*β*_1–42_ male, n = 11; A*β*_1–42_ female, n = 12; A*β*_42–1_ male, n = 7; A*β*_42–1_ female, n = 6) spent the same amount of time performing the different analyzed behaviors (Fig. 5A): locomotion (treatment effect: F_(2,53)_ = 0.630, *p* = 0.5334; sex effect: F_(1,53)_ = 0.8804, *p* = 0.3523), rearing (treatment effect: F_(2,53)_ = 1.204, *p* = 0.308; sex effect: F_(1,53)_ = 0.6201, *p* = 0.4345) and grooming (treatment effect: F_(2,52)_ = 1.885, *p* = 0.162; sex effect: F_(1,52)_ = 3.998, *p* = 0.051). Climbing behavior exhibited a significant sex effect (F_(1,53)_ = 6.770, *p* = 0.012), showing that female mice tended to climb more than male, regardless of the treatment (F_(2,53)_ = 1.626, *p* = 0.2064). All groups equally improved their performance in the rotarod test (Fig. 5B; vehicle male, n = 8; vehicle female, n = 11; A*β*_1–42_ male, n = 8; A*β*_1–42_ female, n = 9; A*β*_42–1_ male, n = 7; A*β*_42–1_ female, n = 6; time effect: F_(5,294)_ = 3.646, *p* = 0.003), as no differences in the latency to fall off the rod were found between groups along trials (treatment effect: F_(2,258)_ = 1.552, *p* = 0.214; sex effect: F_(1,258)_ = 0.415, *p* = 0.52) nor in the whole session (treatment effect: F_(2,43)_ = 0.36, *p* = 0.6998; sex effect: F_(1,43)_ = 0.0957, *p* = 0.7585). Locomotion was also tested in all groups (vehicle male, n = 8; vehicle female, n = 12; A*β*_1–42_ male, n = 9; A*β*_1–42_ female, n = 12; A*β*_42–1_ male, n = 7; A*β*_42–1_ female, n = 6) using the elevated plus maze. The number of entries into closed (treatment effect: F_(2,48)_ = 0.2235, *p* = 0.8006; sex effect: F_(1,48)_ = 0.03188, *p* = 0.859) and total arms (treatment effect: F_(2,48)_ = 0.01929, *p* = 0.9809; sex effect: F_(1,48)_ = 0.0001, *p* = 0.9899) did not differ significantly between groups due to either treatment or sex (Fig. 5C), indicating similar locomotor activity. Regarding stress-related behaviors, all animals had the same number of entries (Fig. 5C; treatment effect: F_(2,48)_ = 1.034, *p* = 0.3633; sex effect: F_(1,48)_ = 0.224, *p* = 0.6381) and time spent in open arms (treatment effect: F_(2,48)_ = 0.6591, *p* = 0.5219; sex effect: F_(1,48)_ = 0.02385, *p* = 0.8779) in the elevated plus maze, and they all had the same immobility time (Fig. 5D; vehicle male, n = 8; vehicle female, n = 12; A*β*_1–42_ male, n = 9; A*β*_1–42_ female, n = 12; A*β*_42–1_ male, n = 7; A*β*_42–1_ female, n = 6; treatment effect: F_(2,48)_ = 0.05294, *p* = 0.9485; sex effect: F_(1,48)_ = 0.1535, *p* = 0.6969) in the tail suspension test, suggesting that the treatment did not increase depression- nor anxiety-like behaviors.

Thus, this data confirmed that the overall health status and locomotor function were uniform among the different treated groups and, therefore, all learning and memory impairments observed in this work were due to a specific hippocampal disruption caused by A*β*_1–42_.

### A*β*_1–42_ inhibits ex vivo LTP similarly in both female and male mice

Given the similar deleterious effects of A*β*_1–42_ on hippocampal-dependent learning and memory processes in both sexes in the present amyloidosis model, we wondered whether excitability, presynaptic function, and short- and long-term plasticity were affected, since they are the underlying physiological mechanisms of those cognitive capabilities. To pair it with the behavioral tasks, electrophysiological recordings were carried out 1–17 days post-*icv.* injection of A*β*_1–42_ or controls: A*β*_42–1_ and vehicle in a new cohort of mice.

**Fig. 6:**
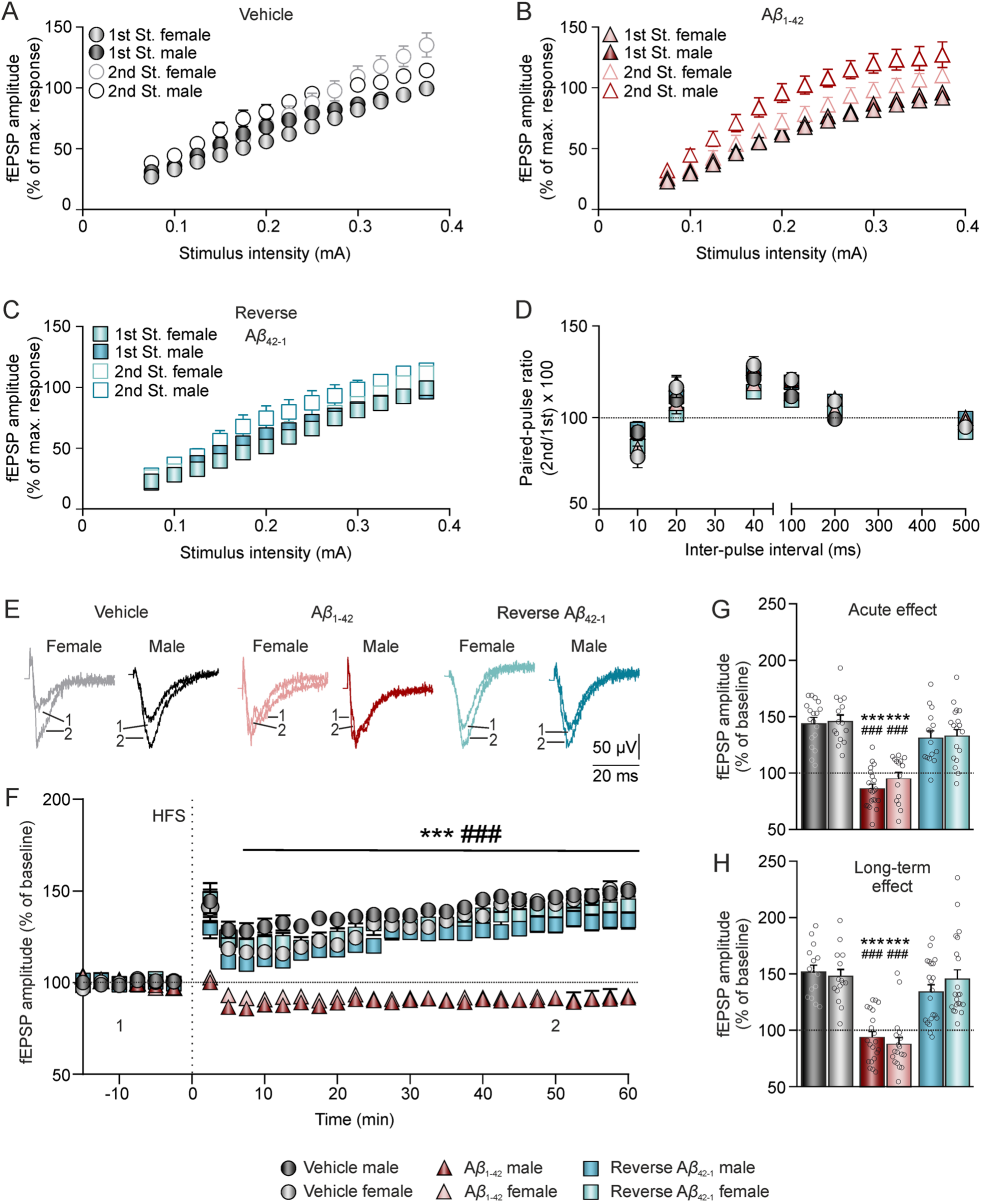
A*β*_1–42_ inhibits ex vivo hippocampal LTP and induces LTD in both female and male mice.** A–C** I/O curve with paired fEPSPs collected at increasing stimulus intensities (from 0.075 to 0.4 mA) from control vehicle (**A**), A*β*_1–42_ (**B**) and A*β*_42–1_ reverse control (**C**) slices, respectively. Data is expressed as a percentage (%) of the maximum amplitude obtained. **D** PPF curve with paired fEPSPs collected at interstimulus intervals of 10, 20, 40, 100, 200 and 500 ms. Data is expressed as mean ± SEM amplitude of the second fEPSP as a percentage of the first [(second/first) × 100] for each inter-pulse interval used. **E** Representative averaged (n = 5) traces of fEPSPs recorded in the CA1 area, collected during the baseline (1) and ≈50 min post-HFS (2) in hippocampal slices from the different groups. **F** Time course of LTP evoked in the CA1 area after HFS in hippocampal slices from the different groups. Recordings were obtained from day 1 to 17 post-*icv.* injection. **G, H** Bars illustrate mean ± SEM fEPSPs amplitude of the last 10 min of the recording, to show acute (**G**; 24–48 h post-*icv.* injection) *vs.* long-term (**H**; 3–17 days post-*icv.* injection) effects on LTP. N (slices) vehicles: males = 6–5 and females = 5–5; N A*β*_1–42_: males = 7–7 and females = 6–7; N reverse A*β*_42–1_: males = 5–7 and females = 6–7. A*β*, amyloid-*β*; HFS, High frequency stimulation; LTP, long-term potentiation; mA, milliamperes; ms, milliseconds; min, minutes. ***p < 0.001 *vs*. vehicle of the corresponding sex; ###*p* < 0.001 *vs*. A*β*_42–1_ of the corresponding sex

Firstly, I/O curves in all groups (Fig. 6A–C) showed a greater amplitude of both the first (vehicle male, n = 7; vehicle female, n = 7; A*β*_1–42_ male, n = 8; A*β*_1–42_ female, n = 5; A*β*_42–1_ male, n = 5; A*β*_42–1_ female, n = 4; F_(2.264,321.436)_ = 929.033, *p* < 0.001, Geisser-Greenhouse’s correction) and the second fEPSP (F_(2.255,209.756)_ = 443.592, *p* < 0.001, Geisser-Greenhouse’s correction) with increasing intensities. No between-group differences were observed in the amplitude of the 1^st^ fEPSP due to either sex (F_(2.264,321.436)_ = 2.638, *p* = 0.066, Geisser-Greenhouse’s correction) or treatment (F_(4.527,321.436)_ = 1.732, *p* = 0.134, Geisser-Greenhouse’s correction). However, a sex-treatment interaction effect was found in the amplitude of the 2^nd^ fEPSP (F_(4.511,209.756)_ = 2.813, *p* = 0.021, Geisser-Greenhouse’s correction), indicating that male animals injected with A*β*_1–42_ exhibited higher amplitudes evoked by the second pulse compared to the other sex-matched groups.

Then, we addressed the short-term plasticity phenomenon, PPF. This protocol is also related to neurotransmitter release and, therefore, allowed us to evaluate the presynaptic functionality following A*β*_1–42_ injection. As shown in Fig. 6D, all groups presented an increased response to the second pulse when the intervals were short (20, 40 and 100 ms), since the ratio between the second and first EPSPs were above 100%, indicating enhanced neurotransmitter release. Nonetheless, neither treatment nor sex caused significant differences at any of the selected intervals (vehicle male, n = 6; vehicle female, n = 6; A*β*_1–42_ male, n = 7; A*β*_1–42_ female, n = 5; A*β*_42–1_ male, n = 3; A*β*_42–1_ female, n = 3; F_(2,83)_ = 0.297, *p* = 0.744 and F_(1,83)_ = 3.144, *p* = 0.08, respectively). This data indicated a normal short-term plasticity and presynaptic vesicle release after the treatment, suggesting that the alterations caused by A*β*_1–42_ injection may preferentially impact the postsynaptic level.

Finally, we measured the effect of A*β*_1–42_ injection on long-term synaptic plasticity applying an HFS protocol after a 15-min baseline from day 1 to 17 post-*icv*. injection (Fig. 6E). Data showed a significant treatment effect (Fig. 6F; vehicle male, n = 11; vehicle female, n = 10; A*β*_1–42_ male, n = 14; A*β*_1–42_ female, n = 11; A*β*_42–1_ male, n = 12; A*β*_42–1_ female, n = 13; F_(2,207)_ = 87.616, *p* < 0.001). *Post-hoc* analysis revealed that the differences were specifically between the A*β*_1–42_ and the two control groups, regardless of sex (sex effect: F_(1,207)_ = 0.092, *p* = 0.762). This inhibition of LTP was observed immediately after the HFS and persisted for at least 60 min afterwards. In fact, fEPSPs post-HFS were slightly below the BL for this group, suggesting that a protocol intended to induce LTP instead induced LTD. Importantly, the control reverse peptide did not affect LTP, indicating the specificity of A*β*_1–42_ peptide´s detrimental effect. Additionally, this study aimed to determine whether the detrimental effect of A*β*_1–42_ on LTP was an acute or long-term effect of *icv.* injection. The results demonstrated that A*β*_1–42_ disrupted LTP both in the short-term (24–48 h post-injection; treatment effect: F_(2,96)_ = 62.30, *p* < 0.001; Fig. 6G) and long-term (3–17 days post-injection; treatment effect: F_(2,105)_ = 49.94, *p* < 0.001; Fig. 6H), irrespective of sex. This finding confirms that *icv.* administration of A*β*_1–42_ has both acute and long-term effects on synaptic plasticity processes. Notably, even at 17 days post-injection, when A*β* levels were similar between A*β*_1–42_ treated and control mice based on our western blot results of amyloid peptide clearance (see Additional file 1 for details), the detrimental effects on synaptic plasticity and memory persisted.

Overall, these results indicate that a single A*β*_1–42_ injection impairs long-term synaptic plasticity, mainly at a postsynaptic level. This effect likely underlies the hippocampal-dependent learning and memory deficits observed in our study in both male and female animals.

## Discussion

The deleterious effect of A*β* on hippocampal-dependent learning and memory has been widely reported [15]. However, the possible differences associated to sex are scarcely explored, notwithstanding the abundance of clinical and epidemiological studies showing greater prevalence, risk and severity of AD in women [33]. Moreover, animal studies, mainly carried out with transgenic mice models, have reported conflicting findings regarding sex differences [35, 38]. Here, we aimed to elucidate whether a single *icv.* administration of A*β*_1–42_, a local model for studying early hippocampal amyloidosis [55, 56], had differential effects on hippocampal function in female and male mice.

Spatial navigation deficits are early indicators of AD [57] and can help differentiate between various types of dementia, i.e., AD and frontotemporal dementia [58], since AD specifically exhibits deficits in spatial working memory compared to other dementia syndromes [59]. In our study, we used the Barnes maze and OLM test to assess spatial memory, and the OF habituation test to evaluate non-associative hippocampal-dependent memory processes. Our results showed a similar impairment in both male and female mice in all forms of spatial learning and memory evaluated, as well as in the exploratory habituation. It is well established that learning and memory deterioration occurs in both AD patients and animal models [25, 60]. Notwithstanding, while transgenic models represent genetic forms of AD and require months to develop cognitive impairments, AD is primarily a sporadic disorder [61]. Moreover, it has been highlighted that AD’s prodromal period can last from 5 years to even decades [26], with the first cognitive symptoms appearing as early as 12 years before the onset of dementia [62]. Therefore, studying early stages of AD pathology is crucial. In agreement with that, *icv.* administration of A*β* mimics the sporadic form of AD during its early stages. Previous works from our group and others have shown that even early amyloidosis caused by a single A*β* injection is sufficient to impair short- and long-term spatial and habituation memory in male rodents [22–24, 30, 31, 59, 63]. The present study extends these findings to demonstrate that working spatial memory is also affected, as it has been shown in transgenic mice models at later stages of the disease [21, 36]. However, this is of special interest in early amyloidosis since working memory deficits can be used to predict the severity of cognitive decline and differentiate patients with mild cognitive impairment (MCI, a prodromal stage of AD) who are at risk of developing this type of dementia [64, 65]. The spatial memory deficits observed in our study are also reflected in the navigation strategies employed by the mice. The vehicle and reverse control groups exhibited a shift towards more spatial strategies over the course of training and tests days, which correlates with improvements in escape latency during the acquisition phase. In contrast, the A*β*_1–42_ treated mice predominantly used a ‘random search’ strategy, leading to minimal improvement in escape latency over time. This alteration in navigation strategy has also been found in AD patients [66, 67] and transgenic mouse models of AD [68, 69]. Importantly, our findings suggest that the detrimental effects of A*β* administration primarily target the dorsal hippocampus region [23] involved in spatial processing [29], while anxiety and depression-like behaviors associated with the ventral hippocampus [70] remain unaffected. Furthermore, it is important to acknowledge the involvement of the hippocampal region in various phases of memory processing and learning, including encoding and retrieval [71], which may be differentially affected by A*β* peptide. Previous data obtained by our laboratory have revealed that *icv.* administration of A*β* impairs the retrieval of previously acquired dorsal hippocampus-dependent memory in male mice [22–24]. However, it remained unexplored how the peptide impacts both the encoding and retrieval phases, particularly in terms of sex differences. Consequently, in this study, we conducted two sets of experiments by administering A*β*_1–42_ before or after the learning sessions to evaluate both encoding and retrieval of memory. Our results demonstrated that *icv.* injection of A*β*_1–42_ disrupts both phases of memory processing, which is consistent with research conducted in AD patients, demonstrating alterations in both processes [72]. However, it should be noted that other phases of memory processing, such as storage and consolidation, may also be impacted, and further investigations are warranted.

On another note, our data did not show any discernible difference between female and male mice neither in the Barnes maze nor in the OF habituation or OLM tests. Some authors had described similar results, showing no sex-dependent differences in transgenic models [38, 39]. Other authors, however, had described the opposite, reporting that female transgenic mice showed more spatial memory deficits than age-matched males [34, 36]. Nonetheless, it is important to note that those mice displayed prominent amyloid plaques as well as neurofibrillary tangles [35] while in our model of early amyloidosis, A*β* is in its soluble form, yet without forming senile plaques. Thus, it could be hypothesized that in the early stages of amyloidosis there may be no sex-dependent alterations, whereas in older animals, with advanced AD pathogenesis, sex becomes a significant factor of disease severity. In this line, studies using 3xTg-AD mouse model, which is widely used to study AD, have demonstrated that male and female mice displayed no memory differences at 4–10 months of age, but female mice performed worse than males at older ages [21, 73, 74]. Interestingly, young females seem to be protected against A*β* toxicity by estrogen, as it promotes a non-amyloidogenic metabolism of APP and has anti-inflammatory properties [33]. This neuroprotection was shown at an age that is considered a good representation of the preclinical stages of AD, which occur more than two decades before disease onset [75]. Later on, when females reach menopause and estrogen levels decline, this neuroprotection diminishes, potentially contributing to the increased susceptibility of post-menopausal women to AD [34]. In fact, human studies have reported a negative correlation between estrogen levels and spatial cognition [76]. Nevertheless, it is crucial to interpret animal studies with caution since the reproductive senescence of female mice do not closely resemble that of menopausal women. To achieve that, ovariectomy or chemically-induced menopause is required [77]. Indeed, a recent study demonstrated that estrogen treatment in ovariectomized 5xFAD mice, another AD mice model, decreased APP and hyperphosphorylated *tau* levels compared to untreated 5xFAD females [78].

Regarding activity-dependent synaptic plasticity mechanisms underlying learning and memory, our data indicates that a single A*β*_1–42_ injection does not alter short-term plasticity nor presynaptic vesicle release but does affect long-term plasticity at postsynaptic level, exhibiting both acute and long-term effects. Several studies concur with the fact that A*β* pathology primarily affects the postsynaptic level [79]. Early deficits in LTP have been associated with A*β* accumulation in the hippocampus of transgenic models, and this weakening in synaptic strength is linked to an inability to use cues in a spatial learning task [80]. The characteristic hyperexcitability caused by A*β* in the dorsal hippocampus may increase the threshold of LTP induction which, following the Bienenstock, Cooper and Munro (BCM) theory, would lead to the induction of LTD as the stimulation fails to properly activate postsynaptic neurons [81]. Moreover, A*β* has been found to indirectly partially unblock synaptic NMDA receptors [82], which are known to make an essential contribution to spatial working memory processing [83]. A*β* also activates metabotropic glutamate receptors [84], which result in increased internalization of AMPA receptors and a shift in signaling cascades toward pathways involved in the induction of LTD and synaptic loss [30, 85, 86]. This effect, along with the BCM theory, could partially explain why our female and male A*β*_1–42_ treated animals displayed LTD instead of the expected LTP following the HFS protocol. In this line, recent studies also showed an HFS-induced LTD both in vivo [24] and ex vivo [23, 59] in male mice following A*β*_1–42_ *icv.* injection. This imbalance in hippocampal synaptic plasticity processes could underlie the observed deficits in hippocampal-dependent learning and memory deficits in both male and female animals, as both LTP and LTD have been shown to play a role in various stages of memory processing, including encoding and retrieval [23, 87]. Interestingly, LTD, which appears to be increased in AD, is mainly associated with habituation to a novel environment [88], a non-associative type of learning that was found to be altered in our mice. The shift in the excitability threshold for LTP/LTD induction caused by A*β* might be partially ruled by G-protein-gated inwardly rectifying potassium (GIRK) channels [23, 89], whose main role in the hippocampus is to maintain the inhibitory tone. Indeed, it has been found that their activation with the selective opener ML297 is able to rescue LTP and associated learning and memory processes in this model of early AD amyloidosis [23, 24, 31]. Additionally, GIRK2 subunit expression is downregulated in different models of AD [90, 91], while training in an hippocampal dependent task normalizes its protein level [91]. Once again, no differences between male and female mice were found, as expected, since there were no sex-dependent changes in memory assessments. Remarkably, our synaptic plasticity results align with our behavioral memory experiments, as the impairment of LTP lasted for the same duration as the observed memory deficits (i.e., up to 17 days after *icv.* injection). This highlights the importance of this A*β icv.* administration model to study early acute-induced AD for up to 2 weeks [55, 56]. In line with this, other authors have shown progressive degeneration following A*β*_1–42_ intrahippocampal administration that persists for up to a month [92–94], and *icv*. administration of A*β* mainly diffuses to the dorsal hippocampus [23].

### Perspectives and significance

Despite the growing number of publications studying the sex-related differences underlying the pathogenesis of AD, data regarding females remains inconsistent. In this regard, our study contributes to this body of knowledge by providing systematic evidence of similar hippocampal deficits caused by A*β*_1–42_ in both male and female mice. This validation of our murine model of early amyloidosis offers significant opportunities and opens numerous research perspectives for future research into the amyloid-related pathogenesis and treatment of AD in both sexes.

## Conclusions

In summary, our results indicate that a single A*β*_1–42_ *icv*. injection leads to similar and robust habituation and spatial working, short- and long-term memory impairments as well as paired LTP inhibition and LTD facilitation in both sexes. Furthermore, the cognitive and synaptic alterations were long-lasting (observed up to 17 days after treatment), evidencing the convenience of using this in vivo mouse model to study early acute stages of amyloidosis regardless of the experimental subject’s sex.

### Supplementary Information

**Additional file 1:** A*β*_1–42_ oligomers are found in the hippocampus 1 and 24 hours after a single* icv.* injection.

## Data Availability

The datasets used and/or analyzed during the current study are available from the corresponding author on reasonable request.
