# Supplementary material for: Systematic characterization of a non-transgenic Aβ1–42 amyloidosis model: synaptic plasticity and memory deficits in female and male mice
Source: Biol Sex Differ. 2023 Sep 16;14:59. doi: 10.1186/s13293-023-00545-4 (PMC10504764; doi:10.1186/s13293-023-00545-4)
Supplement: Supplementary file 1 — Additional file 1: Aβ1–42 oligomers are found in the hippocampus 1 and 24 hours after a single icv. injection. [file 13293_2023_545_MOESM1_ESM.pdf]

Additional file 1. **A*β*1-42 oligomers are found in the hippocampus 1 and 24 hours after a single *icv.* injection**

Western blot protocol

Since behavioral and electrophysiological experiments were carried out from 1 hour to 17 days post-*icv*. A$\beta_{1\text{-}42}$ administration, Western blot was performed to determine the possible A*β* oligomers clearance.

Briefly, hippocampal tissue samples at different time points (1 h, 24 h and 17 days; n = 4-7 per group) were homogenized in ice-cold RIPA lysis buffer (50 mM Tris-HCl pH 7,4; 150 mM NaCl; 0,1% Tx100; 0,5% sodium deoxycholate; 0,1% SDS). Equal amounts of protein (30 µg) were mixed with Laemmli buffer (Bio-rad, USA), then loaded on an SDS-PAGE gel and subjected to electrophoresis. Proteins were transferred to nitrocellulose membranes (Bio-rad, USA) using a transblot apparatus (Bio-Rad, USA). Membranes were blocked with 5% dried skimmed milk powder in Tween-PBS for 1 h to prevent non-specific binding and incubated with the primary antibody against A$\beta_{1\text{-}42}$ (rabbit monoclonal, 1/1000; Abcam, UK) overnight at 4 ºC. After washing, antirabbit HRP-conjugated secondary antibody was added at a dilution of 1/5000 and incubated for 1 h. Blots were washed again, incubated in enhanced chemiluminescence reagent (ECL Prime; Bio-rad, USA), and developed using the G:BOX Chemi XX6 gel documentation system (Syngene, India). Expected molecular weights are 4 kDa for monomers, ≈ 50 kDa for oligomers and higher molecular weights (100-200 kDa) for insoluble fibrils (55). For blot quantification, density of the ≈ 50 kDa band was determined using ImageJ software (ImageJ, USA).

A primary antibody against *β*-actin (mouse monoclonal, 1/5000, 42 kDa; Sigma-Aldrich, USA) and appropriate secondary antibody (antimouse HRP-conjugated, 1/5000) was used as a loading control. Values were expressed as the ratio of the protein of interest/*β*-actin and in percentage of the control group (100%).

A$\beta_{1\text{-}42}$ clearance after a single *icv.* injection

Our results revealed that 1 hour after treatment, both male and female treated with A$\beta_{1\text{-}42}$ exhibited significantly higher levels of the peptide in the hippocampus compared to vehicle-treated mice (Figure S1; treatment effect: $F_{(1,19)} = 52.59$, $p < 0.0001$; sex effect: $F_{(1,19)} = 0.8533$, $p = 0.3672$), confirming successful deposition of the peptide

following a single *icv.* injection. The increased presence of Aβ_{1-42} in the hippocampus was still evident at 24 hours post-injection (Figure S1; treatment effect: $F_{(1,19)} = 9.582$, $p < 0.006$; sex effect: $F_{(1,19)} = 0.1458$, $p = 0.7068$). However, by 17 days after *icv.* injection (corresponding to the last day of behavioral and electrophysiological analysis in our study), Aβ_{1-42} levels were comparable between the treatment and control groups (Figure S1; treatment effect: $F_{(1,14)} = 0.08071$, $p < 0.7805$; sex effect: $F_{(1,14)} = 0.6543$, $p = 0.4321$). This indicates that by day 17 post-*icv*. administration, clearance of the peptide has occurred, although the detrimental effect on synaptic function persists, as demonstrated by the memory and electrophysiological deficits observed.

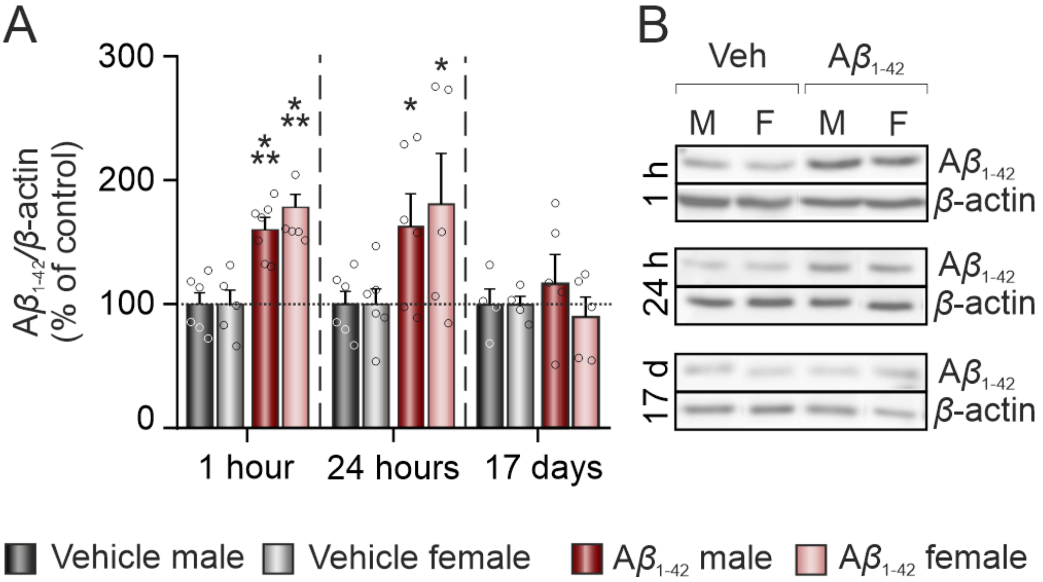

Figure S1. Hippocampal Aβ_{1-42} expression at different time points after *icv.* treatment. (A) Relative expression of Aβ_{1-42} in vehicle and Aβ_{1-42} treated mice 1 hour, 24 hours and 17 days post-*icv*. administration. Data is expressed as the mean ± SEM and as percentage (%) of the controls. **(B)** Representative western blots of Aβ_{1-42} and β-actin, as loading control, in each group. N vehicles: males = 4-6 and females = 4-6; N Aβ_{1-42}: males = 5-7 and females = 5. Aβ, Amyloid-β; d, day; F, female; h, hour; *icv*., intracerebroventricular; M, male; veh, vehicle. * $p < 0.05$, *** $p < 0.001$ *vs*. vehicle of the corresponding sex and time point.
